# Role of the G-Protein-Coupled Receptor Signaling Pathway in Insecticide Resistance

**DOI:** 10.3390/ijms20174300

**Published:** 2019-09-03

**Authors:** Ting Li, Nannan Liu

**Affiliations:** Department of Entomology and Plant Pathology, Auburn University, Auburn, AL 36849, USA

**Keywords:** G-protein coupled receptor regulation pathway, insecticide resistance, *Sf9* cell

## Abstract

The G-protein-coupled receptor (GPCR) regulated intracellular signaling pathway is known to be involved in the development of insecticide resistance in the mosquito, *Culex quinquefasciatus*. To elucidate the specific role of each effector in the GPCR regulating pathway, we initially expressed a GPCR, G-protein alpha subunit (Gαs), adenylate cyclase (AC), and protein kinase A (PKA) in insect *Spodoptera frugiperda* (*Sf9*) cells and investigated their regulation function on cyclic AMP (cAMP) production and PKA activity. GPCR, Gαs, and AC individually expressed *Sf9* cells showed higher cAMP production as the expression of each effector increased. All the effector-expressed cell lines showed increased PKA activity however. Moreover, *Sf9* cytochrome P450 gene expression and cell tolerance to permethrin were examined. The relative expression of CYP9A32gene in *Sf9* cells tested was significantly increased in all effector-expressed cell lines compared to a control cell line; these effector-expressed cell lines also showed significantly higher tolerance to permethrin. Inhibitor treatments on each effector-expressed cell line revealed that Bupivacaine HCl and H89 2HCl robustly inhibited cAMP production and PKA activity, respectively, resulting in decreased tolerance to permethrin in all cell lines. The synergistic functions of Bupivacaine HCl and H89 2HCl with permethrin were further examined in *Culex* mosquito larvae, providing a valuable new information for mosquito control strategies.

## 1. Introduction

G-protein-coupled receptors (GPCRs) are cell surface, membrane-binding proteins that are responsible for signal transmission through extracellular signal binding to activate and regulate intracellular factors. Both the constitutive and spontaneous activities of GPCRs are critically involved in cell signaling responses [1], providing useful opportunities for receptor pharmacology research [2,3]. Active GPCRs transduce signals to heterotrimeric guanine nucleotide-binding proteins (G proteins) that activate or inhibit intracellular factors (e.g., adenylyl cyclase—AC, phospholipase, or ion channels) to elicit a cellular biological response [4]. The cell line-based expression system is favorable for functional studies of the constitutive activity of GPCRs and their downstream cascades [2,3]. Baculorvirus-insect cell expression systems have been widely utilized to produce foreign proteins in insect cells for further functional examination [5] as they not only produce an abundance of GPCRs in a short amount of time (72 h post-infection) [6], but can also be used to build a cell line of GPCR expression for functional identification of intracellular cascades [7].

In the last decade, many studies have confirmed that GPCRs play a crucial role in regulating insect physiological processes such as development, behavior, metabolism, and reproduction. These conserved intracellular pathways are present in several insect species. Because of the importance of functional GPCRs [8] and their unique fingerprint sequences [9], they have often been considered as potential targets for environmentally friendly insecticides for pest control [10]. Recent research has shown that GPCRs and their intracellular effectors (G-protein alpha subunit—Gαs, adenylate cyclase—AC, and protein kinase A—PKA) are involved in the development of insecticide resistance through regulating resistance-related cytochrome P450 gene expression in the mosquito, *Culex quinquefasciatus* [11,12,13]. Injecting cAMP production inhibitor into mosquito larvae lowered the mosquitoes’ resistance to insecticide and suppressed the expression of downstream effectors, in this case PKA and P450 genes, indicating the importance of cAMP in the GPCR regulation pathway and hence the development of insecticide resistance in mosquitoes [11].

This study focuses on the expression of the mosquito GPCR, Gαs, AC, and PKA in insect *Sf9* cells via baculovirus-mediated insect expression in order to investigate the specific function of each effector in insecticide resistance and the P450-expressed regulation of insect cells, as well as their complex connection via second messenger (cAMP) and PKA activity. The findings of this study are expected to not only lead to exciting new insights into intracellular cascades in insecticide resistance, but also to provide useful information that will support the development of novel strategies and/or insecticides for pest control and resistance management in the future.

## 2. Results

### 2.1. Effect of Gene Expression Internalization on cAMP Signaling

**Figure 1:**
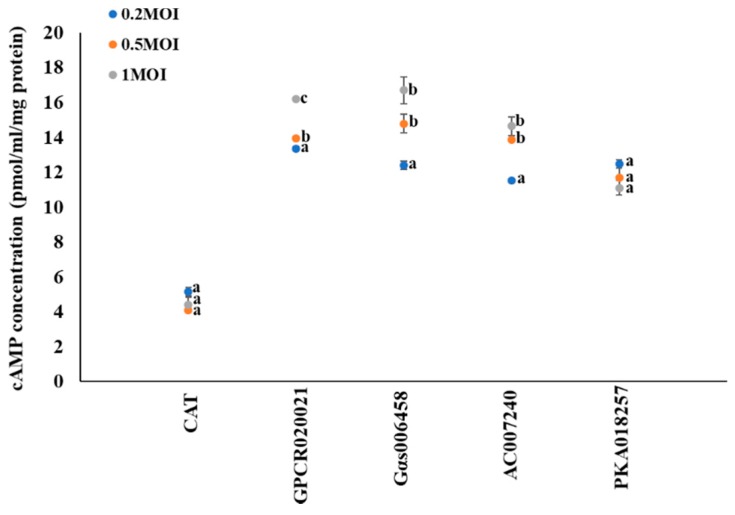
Gene expression associated cyclic AMP (cAMP) production in *Sf9* cell lines. The *x*-axis represents *Sf9* cells infected with recombinant virus of specific genes following the required multiplicity of infection (MOI = 0.2, 0.5, and 1); the y-axis represents the dynamic changes in the cAMP concentration (pmol/mL/mg protein). The blue, orange, and grey solid dots represent multiplicity of infection as 0.2, 0.5, and 1, respectively. The results are shown as the mean ± S.E (*n* ≥ 3). Significant differences (*p* < 0.05) of the cAMP concentrations among the different MOI of gene expression are represented by different alphabetic letters (i.e., a, b, or c), which mean significances among samples.

Previous studies have shown that cell signaling effectors of GPCR–Gαs–AC–PKA–P450 link up to form a functional transduction pathway in *Culex* mosquitoes [11,12,13]. To further investigate the involvement of cAMP in this regulation pathway, we tested the cAMP production in gene expression cell lines. We tested the dynamic changes of cAMP concentrations that followed the increased multiplicity infection of recombinant virus with specific gene expression in cell lines. CAT expression cells served as control. No significant changes of the cAMP concentrations in CAT expression cells (~4 pmol/mL/mg protein) were observed (Figure 1). In the GPCR020021 expressed cell line, the cAMP concentrations significantly increased from 13 to 16 pmol/mL/mg protein following the infection of recombinant virus from 0.2 to 1 MOI (Figure 1). In the Gαs006458 expressed cell line, the cAMP concentrations significantly increased from 12 pmol/mL/mg protein (MOI = 0.2) to 17 pmol/mL/mg protein (MOI = 1) (Figure 1); the same was true for the AC007240 expression cell line, where cAMP concentrations significantly increased from 11 to 14 pmol/mL/mg protein following MOI increase from 0.2 to 1 (Figure 1). In contrast, the results for a cAMP downstream regulation effector, the PKA018257 expression cell line, showed that the cAMP concentrations had no significant changes among all recombinant virus infected cells, although the cAMP concentration was higher in PKA018257 expression cells than that of control CAT expression cells (Figure 1).

### 2.2. Dynamic Changes of the PKA Activity in Gene Expression Cell Lines

**Figure 2:**
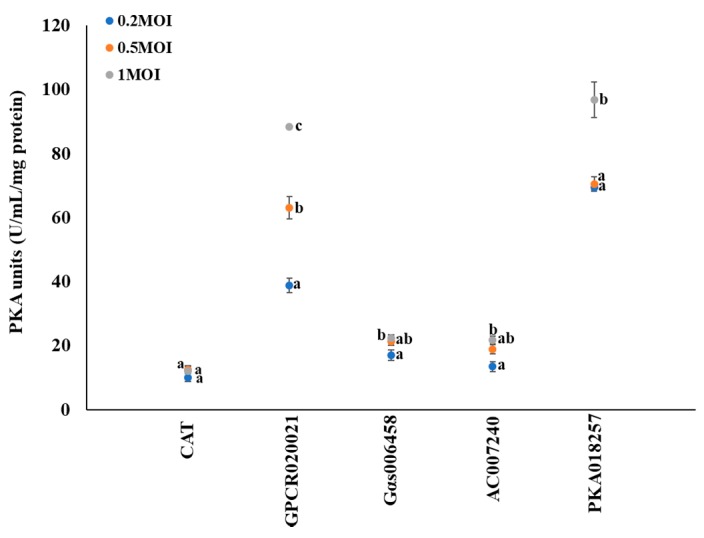
Gene expression associated protein kinase A (PKA) activity in *Sf9* cell lines. The x-axis represents *Sf9* cells infected with recombinant virus of specific genes following the required multiplicity of infection (MOI = 0.2, 0.5, and 1); the y-axis represents the dynamic changes in the PKA activity (U/mL/mg protein). The blue, orange, and grey solid dots represent multiplicity of infection as 0.2, 0.5, and 1, respectively. The results are shown as the mean ± S.E. (*n* ≥ 3). Significant differences (*p* < 0.05) of the PKA activities among the different MOI for gene expression are represented by different alphabetic letters (i.e., a, b, or c), which mean significances among samples.

To investigate the involvement of PKA activity in this regulation pathway, the dynamic changes of PKA activity were observed following the multiplicity infection of recombinant virus with different gene expression in cell lines. CAT expression cell line served as control. There were no significant changes of PKA activity (~10 U/mL/mg protein) in CAT expression cells (Figure 2). Following the increased expression of GPCR020021 in *Sf9* cells, the PKA activity dramatically increased from 40 U/mL/mg protein at MOI = 0.2 to 60 U/mL/mg protein at MOI = 0.5, and 90 U/mL/mg protein at MOI = 1.0. In the Gαs006458 expression *Sf9* cells, the PKA activity significantly increased from 17 to 22 U/mL/mg protein as the MOI ratio rose from 0.2 to 1.0, but there was no significance of PKA activity between MOI ratio of 0.2 and 0.5. In the AC007240 expression *Sf9* cells, PKA activity significantly increased from 14 to 22 U/mL/mg protein as the MOI rose from 0.2 to 1.0, however, no significance of PKA activity between MOI ratio of 0.2 and 0.5 was detected. In the PKA018257 expression *Sf9* cells, the PKA activity significantly increased from 70 to 95 U/mL/mg protein as the MOI rose from 0.2 to 1, as well as the PKA activity showed similar levels as ~70 U/mL/mg at MOI ratio of 0.2 and 0.5 (Figure 2). In addition, the PKA activity showed significant variation among all cell lines. GPCR020021 and PKA018257 cell lines showed the highest PKA activity (around ~90 U/mL/mg) at the MOI = 1, whereas, Gαs006458 and AC007240 cell lines had the PKA activity of 22U/mL/mg at MOI = 1.

### 2.3. Regulation Function of the Effectors on P450 Gene Expression in Sf9 Cells

**Figure 3:**
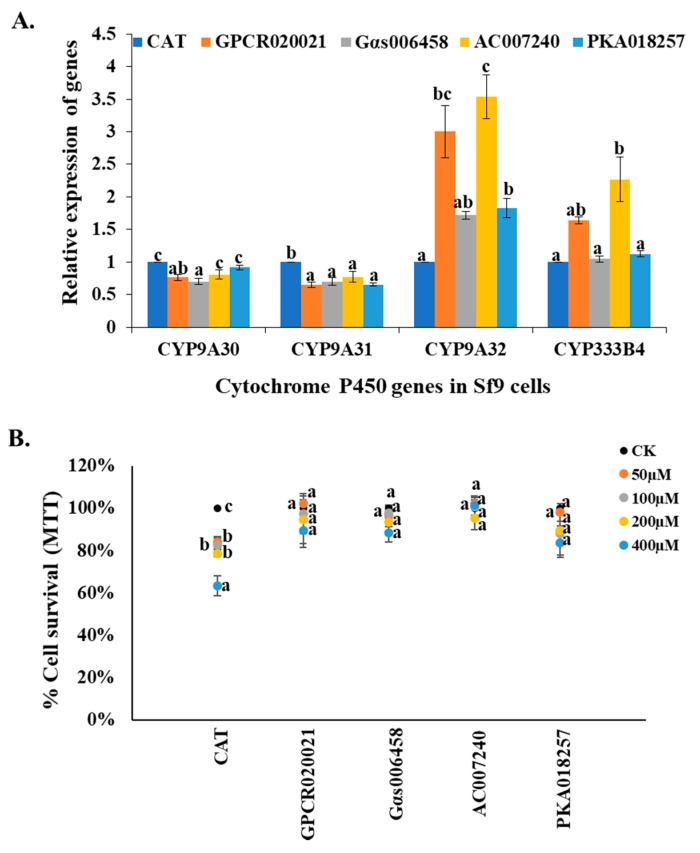
Influence of the effectors on the P450 gene expression and insecticide resistance of *Sf9* cell lines. (**A**) Patterns in the expression of four P450 genes (*CYP9A30*, *-A31*, -*A32*, *CYP333B4*) in the control cell line (CAT expression) and each of the effector expression cell lines (GPCR020021, Gαs006458, AC007240, PKA018257) using qRT-PCR. The relative expression of the P450 genes in the *Sf9* cell lines are shown relative to their expression in the control. (**B**) The effector expression of *Sf9* cells and control cells exposed to serial concentrations of permethrin (50, 100, 200, and 400 µM) using MTT assay. The x-axis represents specific effector expression of *Sf9* cells following the treatment with serial concentrations of permethrin and acetonitrile that served as the control; the y-axis represents the percentage of cell survival ratio under permethrin treatment. The dark, orange, grey, yellow, and blue solid dots represent control and different concentrations of permethrin as acetonitrile alone, 50, 100, 200, 400 μM, respectively. The results are shown as the mean ± S.E (*n* ≥ 3). Significant differences (*p* < 0.05) in the percentage cell survival ratio among the different gene expression cell lines are represented by different alphabetic letters (i.e., a, b, or c), which mean significances among samples.

Previous studies have revealed that GPCR/Gαs/AC/PKA are involved in the regulation of resistance-related P450 gene expression in *Culex* mosquitoes [11,12,13]. In another study, the relative expression of 4 P450 genes (*CYP9A30*, *-A31*, *-A32*, and *CYP333B4*) could be induced by insecticides in *Sf9* cells indicating they may be involved in insecticide resistance [14]. To test the correlation between these regulation effectors and the P450 gene expression in *Sf9* cell lines, we detected the relative expression changes of *CYP9A30*, *-A31*, *-A32*, and *CYP333B4* genes in GPCR020021, Gαs006458, AC007240, and PKA018257 expression cell lines. The results showed one out of four P450 genes (*CYP9A32*) was significantly increased expression in all the cell lines in comparison with the control cell line (CAT) (Figure 3A). The relative expression of *CYP333B4* gene was significantly increased in GPCR020021 and AC007240 cell lines (Figure 3A). However, no significant changes of *CYP9A30* and *CYP9A31* gene expression were observed in all cell lines (Figure 3A). Since these P450 genes have been hypothesized to perhaps play roles in insecticide resistance, we then examined the cytotoxicity of permethrin to the GPCR020021, Gαs006458, AC007240, and PKA018257 expression cell lines, as well as the CAT gene expression cells, which again served as the control. The cell survival percentages of each permethrin concentration treatment were calculated in comparison with that of 0.1% acetonitrile-treated cells. The cell survival ratio decreased significantly, dropping from 85% to 60% as the concentrations of the permethrin rose from 50 to 400 µM in the CAT expression cell line (Figure 3B). However, no significant changes in the cell survival ratio were present in any of the gene expression cell lines under the same concentrations of permethrin treatments as those in the control (Figure 3B). Even at the highest concentration of permethrin (400 µM), the gene-expressed cell lines still exhibited a cell survival ratio of over 85% (Figure 3B).

### 2.4. Effects of cAMP Production Inhibitor on Cell Lines

**Figure 4:**
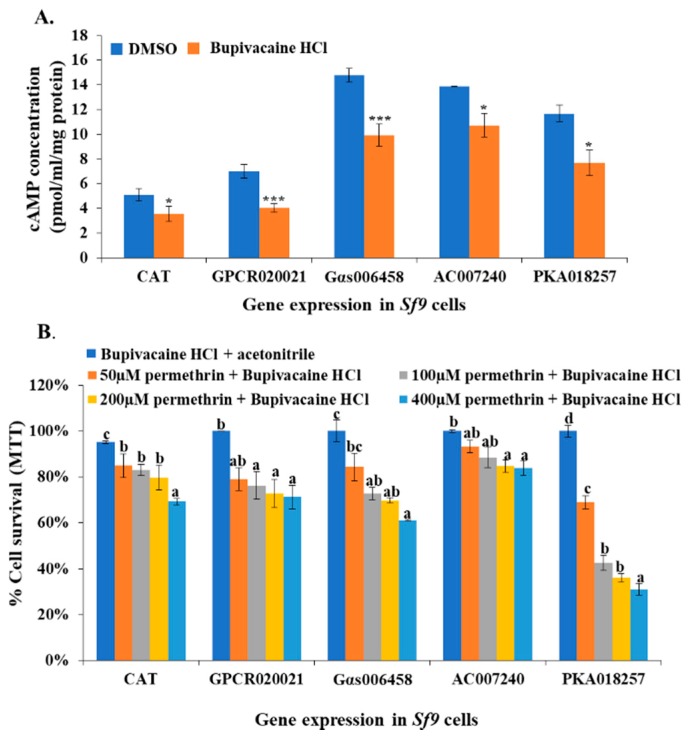
cAMP production inhibitor treatment associated cAMP concentration changes and cell resistance to permethrin in *Sf9* cell lines. (**A**) The effector expression of *Sf9* cells (CAT, GPCR020021, Gαs006458, AC007240, and PKA018257) treated with 40 µM cAMP production inhibitor (Bupivacaine HCl in dimethyl sulfoxide (DMSO)). The x-axis represents different effector expressions of *Sf9* cell lines following Bupivacaine HCl or DMSO alone treatments; the y-axis represents the cAMP concentrations (pmol/mL/mg protein). The blue column represents cells treated with DMSO as control; the orange column represents Bupivacaine HCl-treated cells. (**B**) Percentage of cell survival for different effector expressions of *Sf9* cells treated with 40 µM Bupivacaine HCl and a serial of concentrations of permethrin (50, 100, 200, 400 µM). The *x*-axis represents the different effector expression of *Sf9* cell lines following Bupivacaine HCl and permethrin treatments; the y-axis represents the percentage of cell survival ratio. The blue, orange, grey, yellow, and light blue columns represent Bupivacaine HCl plus acetonitrile, 50, 100, 200, and 400 μM permethrin treatments, respectively. Results are shown as the mean ± S.E (*n* ≥ 3). Statistical significance (*p* ≤ 0.05) for the changes in cAMP concentration in Bupivacaine HCl-treated or DMSO-treated effector expression of *Sf9* cell lines is represented by * *p* < 0.05, *** *p* < 0.001; statistical significance (*p* ≤ 0.05) for the percentage of cell survival ratio in the cell lines treated by permethrin and/or Bupivacaine HCl is represented by different alphabetic letters (i.e., a, b, or c), which mean significances among samples.

Bupivacaine HCl is an effective cAMP production inhibitor. It was utilized to treat the GPCR020021, Gαs006458, AC007240, and PKA018257 expression cell lines, and the CAT gene expression cell line (control line), followed by measuring the cAMP concentration in each treatment. The results showed that the cAMP concentration significantly decreased in the Bupivacaine HCl-treated cell lines (~1.5- to 2-fold) in comparison with the cell lines that were treated with the solvent (dimethyl sulfoxide, DMSO, ACS grade, VWR, Suwanee, GA, USA) (Figure 4A). The synergistic effect of Bupivacaine HCl on the toxicity of permethrin insecticide was then tested in different effector expression cell lines and the control cell line. We utilized the Bupivacaine HCl/acetonitrile to treat all cell lines that showed ~100% survival, which means the Bupivacaine HCl at a certain concentration was toxic to *Sf9* cell lines. There was no difference in the chemical effect between Bupivacaine HCl/acetonitrile and DMSO on *Sf9* cell viability among all cell lines, thus, it is not necessary to use DMSO and acetonitrile to treat the cell lines as a negative control. In the control cell line (CAT expression), there was a decrease in the cell survival ratio from 85% to 60% under permethrin treatment (50 to 400 µM) after inhibitor treatment in comparison with cells treated with the Bupivacaine HCl + acetonitrile (control) (Figure 4B). In the GPCR020021 and Gαs006458 expression cell lines, the Bupivacaine HCl-treated cells showed significantly decreased cell survival ratios of ~70% at various permethrin concentrations (100, 200, and 400 µM) in comparison with control (Figure 4B). In the AC007240 expressed cell line treated with Bupivacaine HCl, the cell survival ratio dropped to ~80% in response to 200 and 400 µM permethrin treatment, although there were no significant changes in cell mortality in 50 and 100 µM treatment (Figure 4B). The cAMP inhibitor-treated PKA018257 expression cell line also exhibited a dramatically decreased cell survival ratio (ranging from 70% to 30%, corresponding to 50 to 400 µM permethrin treatments, respectively) in comparison with the control (Figure 4B).

### 2.5. Potent Effects of PKA Inhibitor on Cell Lines

**Figure 5:**
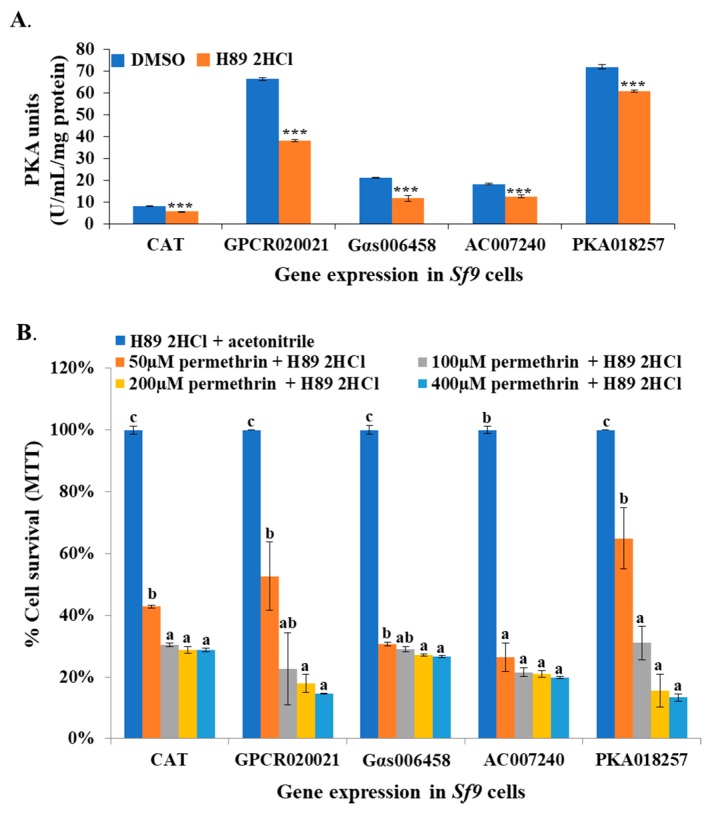
PKA inhibitor treatment associated PKA activity changes and cell resistance to permethrin in *Sf9* cell lines. (**A**) The effector expressions of *Sf9* cell lines (CAT, GPCR020021, Gαs006458, AC007240, and PKA018257) treated with 60 µM PKA activity inhibitor (H89 2HCl). PKA activity was measured in different effector expression cell lines with or without H89 2HCl treatment. The x-axis represents different effector expressions of *Sf9* cell lines following H89 2HCl or DMSO treatments; the y-axis represents the PKA activity (U/mL/mg protein). Blue columns represent cell lines treated with DMSO alone; the orange columns represent H89 2HCl-treated cell lines. (**B**) Percentage of cell survival was measured in different effector expression of *Sf9* cell lines treated with 60 µM H89 2HCl and a serial of concentrations of permethrin (50, 100, 200, 400 µM). The x-axis represents the different effector expression cell lines following H89 2HCl and/or permethrin treatments; the y-axis represents the percentage of cell survival ratio. The blue, orange, grey, yellow, and light blue columns represent H89 2HCl plus acetonitrile, 50, 100, 200, and 400 μM permethrin treatments, respectively. Results are shown as the mean ± S.E (*n* ≥ 3). Statistical significance (*p* ≤ 0.05) for the changes in PKA activity in H89 2HCl-treated or control cell lines are represented by *** *p* < 0.001; for the percentage of cell survival ratio in the effector expression cell lines treated by permethrin and/or H89 2HCl, statistical significance (*p* ≤ 0.05) is represented by different alphabetic letters (i.e., a, b, or c), which mean significances among samples.

To further examine the involvement of PKA activity in the regulation pathway, a universal PKA inhibitor (H89 2HCl) was utilized to treat the GPCR020021, Gαs006458, AC007240, and PKA018257 expression cell lines, and the CAT gene expression cell line. The H89 2HCl-treated cell lines all showed significantly decreased PKA activity compared to cell lines that were treated with the solvent (DMSO) (Figure 5A). The synergistic effect of H89 2HCl on the toxicity of permethrin to *Sf9* cells was examined in specific gene expression cell lines and the CAT expression cell line. In the CAT expression cell line, H89 2HCl-treated cells showed significantly increased susceptibility to permethrin (40% cell survival at 50 µM of permethrin and 30% survival at 100, 200, and 400 µM of permethrin) compared to cells treated with the H89 2HCl + acetonitrile (Figure 5B). The GPCR020021, Gαs006458, AC007240, and PKA018257 expression cell lines, all of which were initially tolerant to permethrin treatment, showed dramatically decreased resistance to all concentrations of permethrin, with 60% to 10% cell survival (corresponding to 50 to 400 µM of permethrin treatment, respectively) in comparison with controls (Figure 5B).

### 2.6. Synergistic Effects of cAMP and PKA Inhibitors on the Toxicity of Permethrin in Culex Mosquito Larva

**Figure 6:**
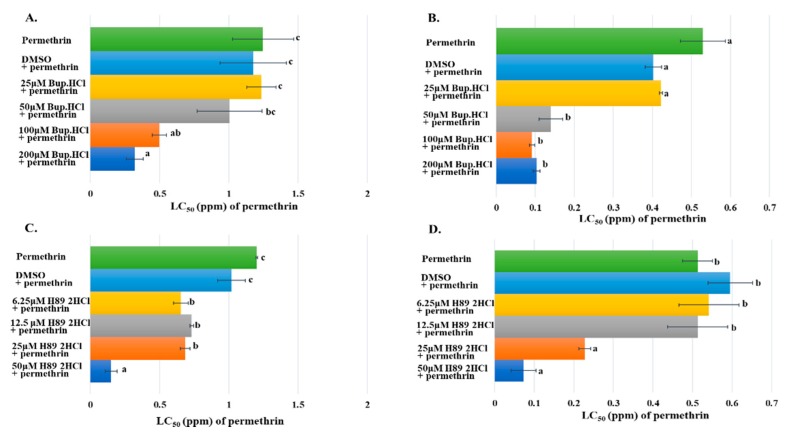
Synergistic roles of Bupivacaine HCl and H89 2HCl to the toxicity of permethrin in insecticide resistant mosquitoes. The x-axis represents LC_50_ (ppm) of permethrin to mosquito larvae; the y-axis represents the permethrin with or without inhibitor treatments to mosquito larvae. (**A**) Early 3rd instar larvae of HAmCq^G8^ treated with serial concentrations of Bupivacaine HCl (Bup.HCl) (25, 50, 100, and 200 µM) and different concentrations of permethrin (0.1, 0.3, 1, and 3 ppm); permethrin alone and permethrin plus DMSO-treated larvae served as controls. (**B**) Early 3rd instar larvae of MAmCq^G6^ treated with serial concentrations of Bupivacaine HCl (Bup.HCl) (25, 50, 100, and 200 µM) and different concentrations of permethrin (0.03, 0.1, 0.3, and 1 ppm); permethrin alone and permethrin plus DMSO-treated larvae served as controls. (**C**) Early 3rd instar larvae of HAmCq^G8^ treated with serial concentrations of H89 2HCl (6.25, 12.5, 25, and 50 µM) and different concentrations of permethrin (0.03, 0.1, 0.3, 1, and 3 ppm); permethrin alone and permethrin plus DMSO-treated larvae served as controls. (**D**) Early 3rd instar larvae of MAmCq^G6^ treated with serial concentrations of H89 2HCl (6.25, 12.5, 25, and 50 µM) and different concentrations of permethrin (0.01, 0.03, 0.1, 0.3 and 1 ppm); permethrin alone and permethrin plus DMSO-treated larvae served as controls. Results are shown as the mean ± S.E (*n* ≥ 3). Statistical significance (*p* ≤ 0.05) for LC_50_ in insecticide resistance mosquito larvae treated by permethrin with or without inhibitor treatments are represented by different alphabetic letters (i.e., a, b, or c), which mean significances among samples.

The synergistic effect of cAMP inhibitor on the toxicity of permethrin was examined in mosquito larvae from two resistant strains. Early 3rd instar larvae of HAmCq^G8^ were treated with a serial of concentrations of Bupivacaine HCl ranging from 25 to 200 µM and simultaneously exposed to serial concentrations of permethrin ranging from 0.1 to 3 ppm. The HAmCq^G8^ larvae showed significantly decreased resistance to permethrin at 100 and 200 µM of Bupivacaine HCl, but lower concentrations of Bupivacaine HCl (25 and 50 µM) had no apparent effect, with treated mosquitoes exhibiting the same resistance level to permethrin to the controls exposed to permethrin alone or DMSO plus permethrin (Figure 6A). The synergistic effect of Bupivacaine HCl was tested in another resistant mosquito strain, MAmCq^G6^, in this case showing significantly decreased resistance to permethrin after treatment with 50, 100, and 200 µM of Bupivacaine HCl in comparison with control groups (Figure 6B). The synergistic effects of a PKA activity inhibitor, H89 2HCl, on the toxicity of permethrin was further confirmed in the resistant mosquito larvae. Serial concentrations of H89 2HCl (6.25, 12.5, 25, 50 µM) used to treat early 3rd instar larvae of HAmCq^G8^ significantly decreased their resistance to permethrin compared with permethrin alone or DMSO plus permethrin treatments (Figure 6C), with the resistance decreasing 7-fold under 50 µM of H89 2HCl treatment in HAmCq^G8^ in comparison to the controls (Figure 6C). A similar function for H89 2HCl was identified in the MAmCq^G6^ mosquitoes, whose resistance to permethrin decreased significantly 3- and 7-fold under 25 and 50 µM treatments of H89 2HCl, respectively (Figure 6D).

## 3. Discussion

Although the underlying mechanisms governing insecticide resistance have been determined in many insect species since the 1950s, the intracellular cascades involved in resistance gene regulation have not been as widely studied [15,16]. Understanding the intracellular regulation pathways in insecticide resistance is a prerequisite if we are to develop new strategies for insect control. Previous studies implicated a regulatory pathway led by GPCRs in the insecticide resistance of mosquitoes by regulating the expression of several P450 genes [11,12] known to metabolize insecticides [17]. The primary function of GPCRs is to govern the active state of the G-protein subunits that trigger diverse intracellular signaling events and cause cell-based physiological responses [18]. In the previous study, we found that GPCRs coordinate the activity of Gαs to govern their down-stream respective gene expressions, including ACs and PKAs, leading eventually to P450 gene up-regulation in *Culex* mosquitoes [13]. To investigate the essential role of each effector and the involvement of the intracellular second message cAMP, as well as the PKA activity in this regulation pathway, we utilized a baculovirus-mediated insect cell expression system to express GPCR, Gαs, AC, and PKA in *Sf9* cells and then examined the cAMP accumulation, PKA activity, and cell viability of insecticide treatments.

The regulation of cAMP signal accumulation is a ubiquitous mechanism responsible for regulating intracellular functions via the GPCR-Gαs-AC-PKA pathway in both vertebrate cells [19,20] and insect cells [21]. The measurement of cAMP concentration has also been extensively utilized to identify the regulatory effectors in intracellular pathways [22]. The findings of the current study show that in GPCR020021, Gαs006458, and AC007240 individually expressed cell lines, the cAMP concentration increases following increased gene expression, indicating the function of each effector in regulating cAMP production. Interestingly, the PKA018257 expressed cell line depressed cAMP levels, suggesting the PKA018257 expression consumes cAMP in insect cells. As a downstream-responding compound of cAMP, PKA activity was then examined in the GPCR020021, Gαs006458, AC007240, and PKA018257 expression cell lines, revealing that the PKA activity increased following individually increased gene expression. All the effector expressed cell lines treated with cAMP production inhibitor and PKA activity inhibitor showed significantly decreased cAMP concentrations and PKA activities in all cell lines, respectively, further confirming the importance and involvement of each effector in this GPCR regulation pathway.

This study investigated the effects of each effector in the GPCR regulation pathway on both P450 gene expression and insecticide resistance in *Sf9* cells. The relative expression of four P450 genes known to be induced by deltamethrin insecticide in *Sf9* cells [14] were characterized in each effector expression cell line. The results showed increased expression of *CYP9A32* in all cell lines in comparison with the control cell line. Further, *CYP333B4* was overexpressed in GPCR020021 and AC007240 cell lines. The other two genes did not show the significant expression changes in cell lines indicating that they may involve in the insecticide susceptibility but not regulated by the GPCR-regulation pathway in our study. An MTT assay conducted to determine the toxicity of permethrin to the GPCR, Gαs, AC, and PKA expression cell lines found a dramatic increase in the tolerance to permethrin in all the cell lines compared to the control cell line, which showed a significantly decreased tolerance to permethrin. However, inhibitors of cAMP production and PKA activity robustly impaired the cell tolerance to permethrin, revealing that these inhibitors act as synergists to boost the toxicity of permethrin, thus further confirming the functional importance of cAMP production and PKA activity in the insecticide resistance of insect cells. Collectively, these results conclusively demonstrate the universal regulatory function of GPCR/Gαs/AC/PKA in insecticide resistance through regulating resistance-related P450 gene expression in insect species such as *Cx. quinquefasciatus* [11,12,13], *D. melanogaster* [11], and *S. frugiperda*. Additionally, the synergistic studies examining the effect of the cAMP production inhibitor and PKA activity inhibitor on the toxicity of permethrin in *Culex* mosquito larvae not only further confirmed the critical function of cAMP and PKA in the insecticide resistance of mosquitoes, but also identified two effective synergists that could potentially be utilized in novel insect control and management methods in the future.

## 4. Materials and Methods

### 4.1. Gene Construction and Cell Culture

The cDNAs for GPCR020021 (Gene ID: CPIJ020021), Gαs006458 (Gene ID: CPIJ006458), AC007240 (Gene ID: CPIJ007240), and PKA018257 (Gene ID: CPIJ018257) were amplified by polymerase chain reaction (PCR) using forward primers containing 4 nucleotides (CACC) prior to the start codon (ATG) that stabilize the PCR product cloning into the Gateway entry vector pENTR/D-TOPO following the procedure described in the pENTR/D-TOPO Cloning Kit manual (Invitrogen, Carlsbad, CA, USA). Details of the primer pairs are listed in Table 1. Constructs were identified by DNA sequencing and subsequently recombined with the C-terminal BaculoDirect^TM^ vector (Invitrogen, Carlsbad, CA, USA) using LR Clonase Enzyme mix (Invitrogen, Carlsbad, CA, USA). The LR reaction was analyzed by PCR with primers of Polyhedrin forward primer and V5 reverse primer according to the manufacturer’s instructions. *Sf9* insect cells were purchased from Invitrogen (Carlsbad, CA, USA) and the *Sf9* cells were grown and maintained in SF 900 III SFM medium (Thermo Fisher Scientific, Waltham, MA, USA) supplemented with 10% FBS (Heat Inactive, Thermo Fisher Scientific, Waltham, MA, USA). The cell lines were cultivated either in monolayers or in suspension at 27 °C, shaken at 120 rpm. Transfections with recombinant virus following the required multiplicity of infection (MOI) were performed in suspension at a cell density of 1.0 × 10^6^ cells/mL. Specifically, GPCR, Gαs, AC, and PKA recombinant with P2 MOI of 0.2, 0.5, and 1 were expressed in *Sf9* cells for 72 h prior to cell lysis preparation. Chloramphenicol acetyltransferase (CAT) expression cells with the same serial MOI served as controls. Cell harvest was determined at the peak of production (72 h post-infection) for tests of cAMP production, PKA activity, and P450 gene expression.

### 4.2. cAMP Measurement

To identify the critical function of cAMP and its connection with each effector in the GPCR regulation pathway, the cAMP production in gene expressed *Sf9* cell lines was examined using the Detect X Direct Cyclic AMP Enzyme Immunoassay Kit (Arbor Assays) following the instructions in the kit manual. 1 × 10^8^ cells/mL were washed by PBS and then directly treated with Sample Diluent for 10 min at room temperature, followed by centrifugation at ~1000 rpm at 4 °C for 15 min, after which the supernatant was assayed. Samples containing cAMP were bound by the cAMP antibody and cAMP-peroxidase conjugate. After 2 h of incubation at room temperature with shaking, the reaction was terminated and the intensity of the generated color at 450 nm was measured via a Cytation 3 imaging reader. Moreover, a cAMP production inhibition test was conducted by treating the recombinant gene expressed cells (MOI = 0.5) with 40 µM Bupivacaine HCl (in DMSO) (CAS no. 18010-40-7, Selleck Chemicals, Houston, TX, USA) [23] for 2 h prior to cell lysate preparation. DMSO alone treatment served as control. cAMP concentrations (pmol/mL) were calculated based on a standard cAMP concentration curve. Total protein concentrations were measured for each sample to confirm the accuracy of the cAMP concentration calculation (pmol/mL/mg protein). Each treatment was repeated at least 3 times with independent infections of the construction baculovirus.

### 4.3. Protein Kinase A Activity Assay

To investigate the relationships linking GPCR–Gαs–AC–PKA, a PKA activity test was performed based on the GPCR, Gαs, AC, and PKA recombinant expression in *Sf9* cells. CAT expression cells served as the control. The Detect X PKA (Protein Kinase A) Activity Kit (Arbor Assays, Ann Arbor, MI, USA) was used to determine the PKA activity following the kit manufacturer’s instructions. Briefly, suspension *Sf9* cells were prepared at a cell density of 1.0 × 10^8^ cells/mL after 72 h expression with recombinant gene (a serial of MOI = 0.2, 0.5, 1) then lysed in Activated Cell Lysis Buffer (Cell lysate buffer, 1 µL/mL PIC, 1 mM PMSF, and 10 mM activated orthovanadate) for 30 min on ice with occasional vortexing. The resulting cell lysate mixture was then centrifuged at 10,000 rpm at 4 °C for 10 min, and the supernatant utilized for PKA assay. Lysate containing PKA was used to phosphorylate the immobilized PKA substrate on a microtiter plate using the kit-provided ATP to produce a phosphor-PKA substrate that could be bound by a specific rabbit antibody and then detected by peroxidase-conjugated Goat-anti-Rabbit. The absorbance of the reaction was determined using a Cytation 3 imaging reader at 450 nm in order to determine the amount of PKA in the samples. To further confirm the involvement of PKA in the GPCR regulatory pathway, recombinant gene-expressed *Sf9* cells were treated with a potent PKA inhibitor (H89 2HCl, CAS no. 130964-39-5, Selleck Chemicals, Houston, TX, USA; Chijiwa et al., 1990). The GPCR, Gαs, AC, PKA expression (MOI = 0.5) *Sf9* cells were pretreated with 60 µM H89 2HCl (in DMSO) at 27 °C for 1 h avoiding light, then 10 µM forskolin (in DMSO) (CAS no. 66575-29-9, Selleck Chemicals, Houston, TX, USA) [24] and 30 µM H89 2HCl were added to the fresh medium 5 min prior to the cell lysis preparation. DMSO alone treatment served as control. The PKA units were measured and calculated according to the standard curve of PKA units (U/mL). Total protein concentrations were measured by a BioRad protein assay and utilized for PKA activity calculation to improve the accuracy of the PKA activity measurement (U/mL/mg protein). Each treatment was repeated at least 3 times with independent infections of the construction baculovirus.

### 4.4. RNA Isolation, cDNA Preparation, and Quantitative Real-Time PCR (qRT-PCR)

Total RNAs were isolated from GPCR020021, Gαs006458, AC007240, PKA018257, and the CAT expression *Sf9* cell lines (MOI = 0.5) using the E.Z.N.A. HP Total RNA Kit (Omega). Prior to cDNA synthesis, genomic DNA was removed from the total RNA using a TURBO DNA-free kit (Ambion, company, Austin, TX, USA). cDNA was synthesized by SuperScript^®^ IV Reverse Transcriptase (Invitrogen, Carlsbad, CA, USA) following the instructions in the kit manual. qRT-PCR was performed using the All-in-One^TM^ qPCR Mix (GeneCopoeia^TM^, Rockville, MD, USA) and CFX^TM^ 96 Real Time system. Each qRT-PCR reaction (25 µL) consisted of SYBR Green master mix, specific primer pairs for four *Sf9* P450 genes [14] (Table 1) at final concentrations of 0.2 µM, and a 1 µg cDNA template from each sample site. The negative control was a “no-template” reaction. The reaction cycle applied the following PCR program: A melting step of 95 °C for 10 min, followed by 40 cycles of 95 °C for 10 s, 60 °C for 20 s, and 72 °C for 15 s. At the end of the final cycle, the temperature was increased from 72 to 95 °C to produce a melting curve with which to assess the specificity of each PCR reaction using Precision Melt Analysis software [25]. All the reactions were run on 3 technical replicates. Relative expression levels of the target genes were analyzed by the 2^−∆∆*C*T^ method [26]. The L-18 and G6PD genes served as an endogenous control [14] (Table 1). Each experiment was repeated on more than 3 independent isolated RNA mosquito samples.

### 4.5. Cell Viability Assay

The relative number of viable *Sf9* cells was measured by a 3-(4,5-dimethylthiazol-2-yl)-2,5 diphenyltetrazolium bromide MTT (Sigma) colorimetric assay [17]. Typically, suspensions of *Sf9* cells after 48 h infection by a construction baculovirus, either GPCR020021, Gαs006458, AC007240, or PKA018257 (MOI = 0.5), were transferred to a 24-well plate (Tissue Culture Treated, CytoOne) at a cell density of 1.0 × 10^6^ cells/mL. CAT-infected *Sf9* cells served as control. The cells were then treated with permethrin (94.34%, supplied by FMC Corp., Princeton, NJ) at a series of concentrations (50, 100, 200, and 400 µM in acetonitrile dilution); cells treated with acetonitrile alone served as solvent controls (CK) in each cell line treatment. The cell survival rate was evaluated by MTT assay after 48 h permethrin treatments. Briefly, the culture medium was removed and 200 µL MTT (5 mg/mL) added to each well. The MTT-treated cells were then incubated avoiding light at 37 °C for 4 h. After incubation, 1 mL of DMSO was added to each well. The absorbance of the resulting solution was determined using a Cytation 3 imaging reader at 540 nm. To test the regulation pathway through cAMP and PKA activity, each effector expression cell line was treated with either 40 µM of the cAMP production inhibitor (Bupivacaine HCl) or 60 µM of the PKA activity inhibitor (H89 2HCl) and simultaneously treated by permethrin, as described above. Inhibitors with acetonitrile was served as control treatment. The cell survival ratio was calculated as (OD value of permethrin and/or inhibitor treated cells/OD value of acetonitrile treated cells) * 100%. Each treatment was repeated at least 3 times with independent infections of the construction baculovirus.

### 4.6. Mosquito Strains and Bioassay

Two insecticide resistant mosquito strains of *Cx. quinquefasciatus,* HAmCq^G8^ and MAmCq^G6^, were utilized in this study. HAmCq^G8^ is the eighth generation of permethrin-selection HAmCq^G0^ offspring that was collected from Mobil County, AL; MAmCq^G6^ is the sixth generation of permethrin-selection MAmCq^G0^ offspring that was collected from Madison County, AL. These two counties are located more than 600 km apart. HAmCq^G8^ and MAmCq^G6^ have obtained ~2700- and ~590-fold higher levels of resistance than lab-susceptible mosquito strains, respectively. To maintain the resistant levels, HAmCq^G8^ and MAmCq^G6^ strains were treated with certain concentrations of permethrin every 6 months [27,28]. The mosquitoes were reared at 25 ± 2 °C under a photoperiod of 12:12 (L:D) h and 70–80% humidity. Female adults were fed cow blood samples (Large Animal Teaching Hospital, College of Veterinary Medicine, Auburn University). To test the synergistic activity of the cAMP production and PKA activity inhibitors on the toxicity of permethrin to mosquitoes, mosquito early 3rd instar larvae were treated with serial concentrations of inhibitors and permethrin. The bioassay method was as described in a previous study [27]. Briefly, inhibitors were diluted from a stock solution (200 µM in DMSO) to create serial concentrations in DMSO (for Bupivacaine HCl these were 200, 100, 50, 25 µM; for H89 2HCl these were 50, 25, 12.5, 6.25 µM). Each bioassay consisted of 30 3rd instar larvae of *Cx. quinquefasciatus* in 6 oz. Sweetheart ice cream cups (Sweetheart Cup Co., Owings Mills, MD, USA) containing regular tap water, inhibitors, and 1% permethrin solutions in acetone at four or five concentrations that yielded >0% and <100% mortality. Serial concentrations of the inhibitors were applied simultaneously with permethrin. Control groups received either permethrin alone or DMSO plus permethrin solution. All tests were performed at 25 °C and replicated at least four times on different days. Mortality was assessed after 24 h. Bioassay data were pooled, and a probit analysis was conducted using Abbott’s correction for control mortality.

### 4.7. Data Analysis

The statistical significances for the cell viability, PKA activity, cAMP production, and gene expression were calculated using a Student’s *t*-test for all 2-sample comparisons and a one-way analysis of variance (ANOVA) for multiple sample comparisons using Statistical Package for the Social Science (SPSS) software with both least significant difference (LSD) and Tukey tests to analyze the significance of the means; a value of *p* ≤ 0.05 was considered statistically significant. All significances were present by alphabetic letters (e.g., a, b, c).

## Figures and Tables

**Table 1:** Oligonucleotide primers used in qRT-PCR and PCR reactions.

Primer Description	Primer Name	Primer Sequence
GPCR020021	GPCR020021 F	5′CACCATGGCATCTTACGCAGCATG3′
	GPCR020021 R	5′TTAAGCCTCGATCTTCTCCGC3′
Gαs006458	Gαs006458F	5′CACCATGGGTTGCTTCGGATC3′
	Gαs006458R	5′CTATAACAGCTCGTATTGCCGCA3′
AC007240	AC007240 F	5′CACCATGTCCCTGTTGCG3′
	AC007240 R	5′TTAGTTCTGCTCCTTGGCGATGC3′
PKA018257	PKA018257F	5′CACCATGGGAAACAACGCAACTTCA3′
	PKA018257R	5′TTAAAATTCTGCAAATTCTTTTGC3′
L-18	L-18 F	5′-CGTATCAACCGACCTCCACT-3′
	L-18 R	5′-AGGCACCTTGTAGAGCCTCA-3′
G6PD	G6PD F	5′-GGCCCTGTGGCTAACAGAAT-3′
	G6PD R	5′-CATCGTCTCTACCAAAAGGCTTC-3′
*Sf*CYP9A30	*Sf*CYP9A30 F	5′-GTCCTGGTGGCTGTGGTATT-3′
	*Sf*CYP9A30 R	5′-GTGCGAAAAATGATCGTGTG-3′
*Sf*CYP9A31	*Sf*CYP9A31 F	5′-ATGCTCGTCTTGGTCTGGTT-3′
	*Sf*CYP9A31 R	5′-CTGCCCATGTTACCGAAGAT-3′
*Sf*CYP9A32	*Sf*CYP9A32 F	5′-ATCATTCGTAAGGGCCAGTG-3′
	*Sf*CYP9A32 R	5′-AAGTGAACGGGACGATTTTG-3′
*Sf*CYP333B4	*Sf*CYP333B4 F	5′-GAATTATGCCGGTGGTGTCT-3′
	*Sf*CYP333B4 R	5′-TAGCGACATGTCTCGGTGAG-3′
